# Preliminary study of confounding factors of elastography and the application of fine-needle aspiration in thyroid nodules with indeterminate elastography

**DOI:** 10.1038/s41598-017-18121-3

**Published:** 2017-12-21

**Authors:** Qiong Wu, Yanhui Qu, Xuedan Zang, Yi Li, Xiaolei Yi, Yan Wang, Bing Hu

**Affiliations:** Department of Ultrasound in Medicine, Shanghai Jiao Tong University Affiliated Sixth People’s Hospital, Shanghai Institute of Ultrasound in Medicine, Shanghai, China

## Abstract

To investigate confounding factors of real-time ultrasound elastography (RTE) and to evaluate the diagnostic performance of ultrasound (US)-guided FNA for thyroid nodules with indeterminate elastography compared with conventional US. This study included 244 nodules with indeterminate elastography caused by several confounding factors (large or small size, deep location, isthmic or paratracheal location, calcification, thyroiditis, conflicting results between conventional US and RTE), and corresponding prevalences of malignancy were calculated. Additionally, conventional US and US-FNA data were collected and compared. The prevalences of malignancy of confounding factors were 74.1%, 75.0%, 73.3%, 46.2%, 27.3%, and 53.2%, respectively. Sonographic features (border, margin, echogenicity, echohomogeneity, and microcalcification) were significantly different between benign and malignant thyroid nodules (p < 0.05), and most of them exhibited good sensitivity but unsatisfactory specificity and accuracy. While US-FNA exhibited better performance with a sensitivity of 96.9%, a specificity of 99.1% and an accuracy of 98.0% in the diagnosis of malignancy. Given that indeterminate RTE is inevitable with a rather high malignant risk due to several confounding factors, our study revealed that US-FNA was a valuable tool in nodules with indeterminate elastography by increasing the detection rate of thyroid malignancy.

## Introduction

Thyroid nodules are increasingly common and can be found in approximately 67% of the general population given the widespread application of ultrasonography (US) imaging techniques^1^, while the incidence of thyroid cancer is up to 5–15%^2,3^. Ultrasound-guided fine-needle aspiration (FNA) is such an accurate and cost-effective tool worldwide that the guidelines from American Thyroid Association (ATA) indicated that it should be the first testing method for the diagnosis of thyroid nodules^4,5^. However, it seems almost impossible to perform FNA for each thyroid nodule with appropriate indications in China, which is a typical country with a large population.

Real-time ultrasound elastography (RTE) is emerging as another effective but non-invasive tool to differentiate malignant from benign thyroid nodules by evaluating tissue stiffness with high sensitivity and specificity^6^. Moreover, several studies have specifically evaluated the utility of RTE in refining the diagnosis of thyroid nodules with indeterminate or non-diagnostic cytology, and the application of the combination of US and RTE was helpful in relevant managements^7–10^. RTE has been widely applied in many countries, including China, to date^6,11,12^. Using our institution as an example, RTE is typically applied with excellent performance in indeterminate nodules from conventional US examination^11^. However, some uncertainties regarding indeterminate diagnosis are associated with RTE and caused by several factors, including rather large or small size, deep location, isthmic or paratracheal position, calcification, and background of thyroiditis^13^. In addition, conflicting results between conventional US and RTE features are occasionally inexplicable in our previous experiences.

Given that the above factors may cause a false positive or negative result, the diagnosis from RTE would not be reliable. We consider the diagnosis to be indeterminate elastography, which could not provide a definite benign or malignant judgement. Then, what to do next? A diagnostic operation is a feasible solution but not optimal. To the best of our knowledge, few studies have assessed the optimal course of action for indeterminate cases based on RTE. Therefore, the purpose of our study was to investigate the common confounding factors of RTE and evaluate the diagnostic performance of further ultrasound (US)-guided FNA for thyroid nodules with indeterminate elastography compared with conventional US.

## Materials and Methods

### Patients

**Figure 1:**
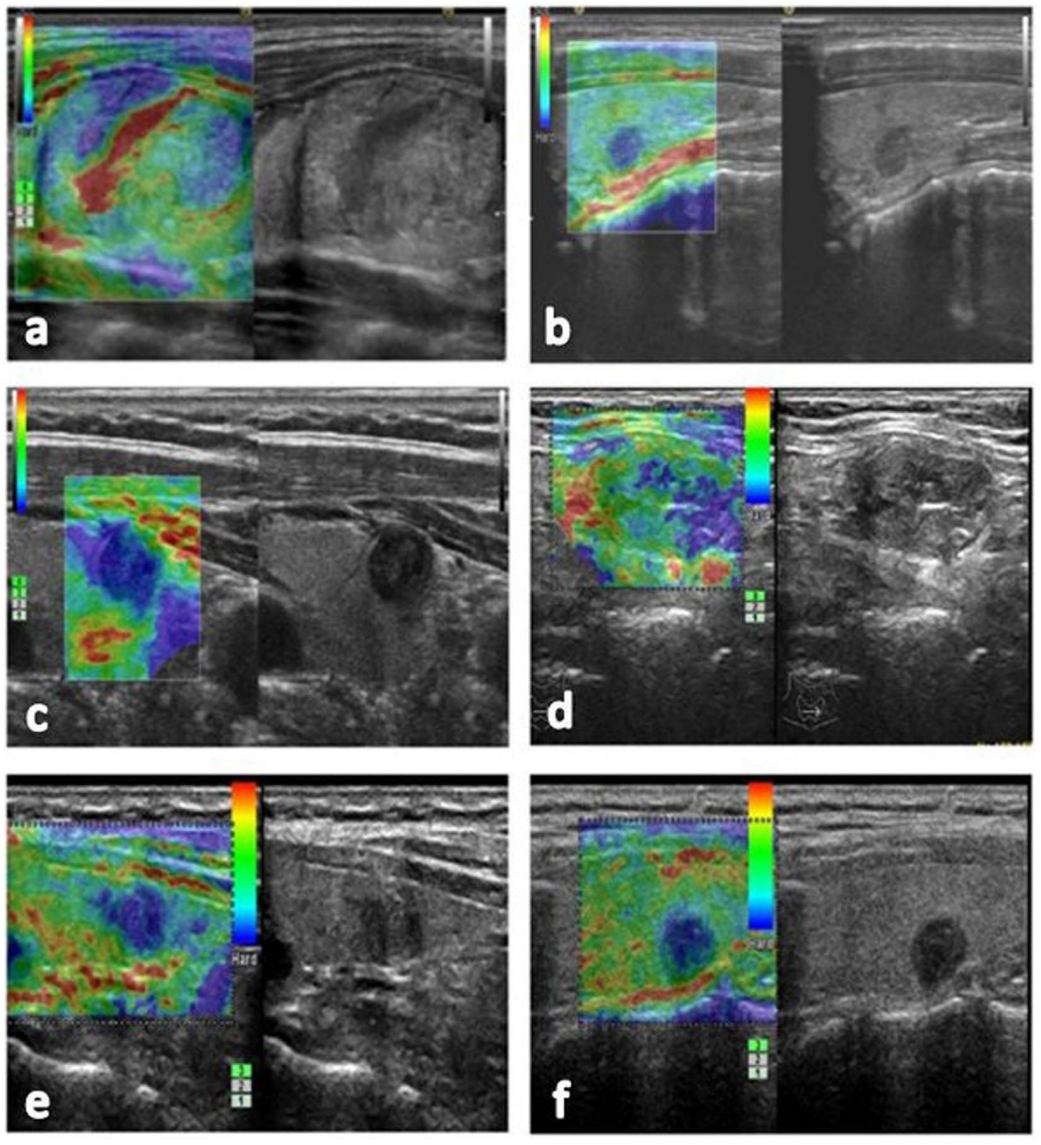
Different nodules with indeterminate elastography. (**a**) Elastography revealed an inhomogeneous distribution of blue and green in a 3.5 cm nodule without much reference parenchyma, which indicated indeterminate elastography. This nodule was confirmed as benign by both fine-needle aspiration and histopathology. (**b**) Elastography revealed homogeneously blue in a deep-located nodule of 0.5 cm, which indicated malignancy. This nodule was confirmed as papillary thyroid carcinoma by both fine-needle aspiration and histopathology. (**c**) Elastography revealed homogeneously blue in an isthmic nodule of 1.1 cm, which indicated malignancy. This nodule was confirmed as benign by fine-needle aspiration. (**d**) Elastography showed half blue and green in a 2.3 cm nodule with macro-calcification, which indicated indeterminate elastography. This nodule was confirmed as benign by fine-needle aspiration and histopathology. (**e**) Elastography showed mostly blue in a 1.2 cm nodule with obscure border, which indicated malignancy. This nodule was confirmed as subacute thyroiditis by fine-needle aspiration though the patient remained asymptomatic at diagnosis. (**f**) Elastography showed mostly blue in a 0.9 cm nodule with clear border and regular margin, which indicated malignancy. Fine-needle aspiration revealed a benign nodule with old hemorrhage.

**Figure 2:**
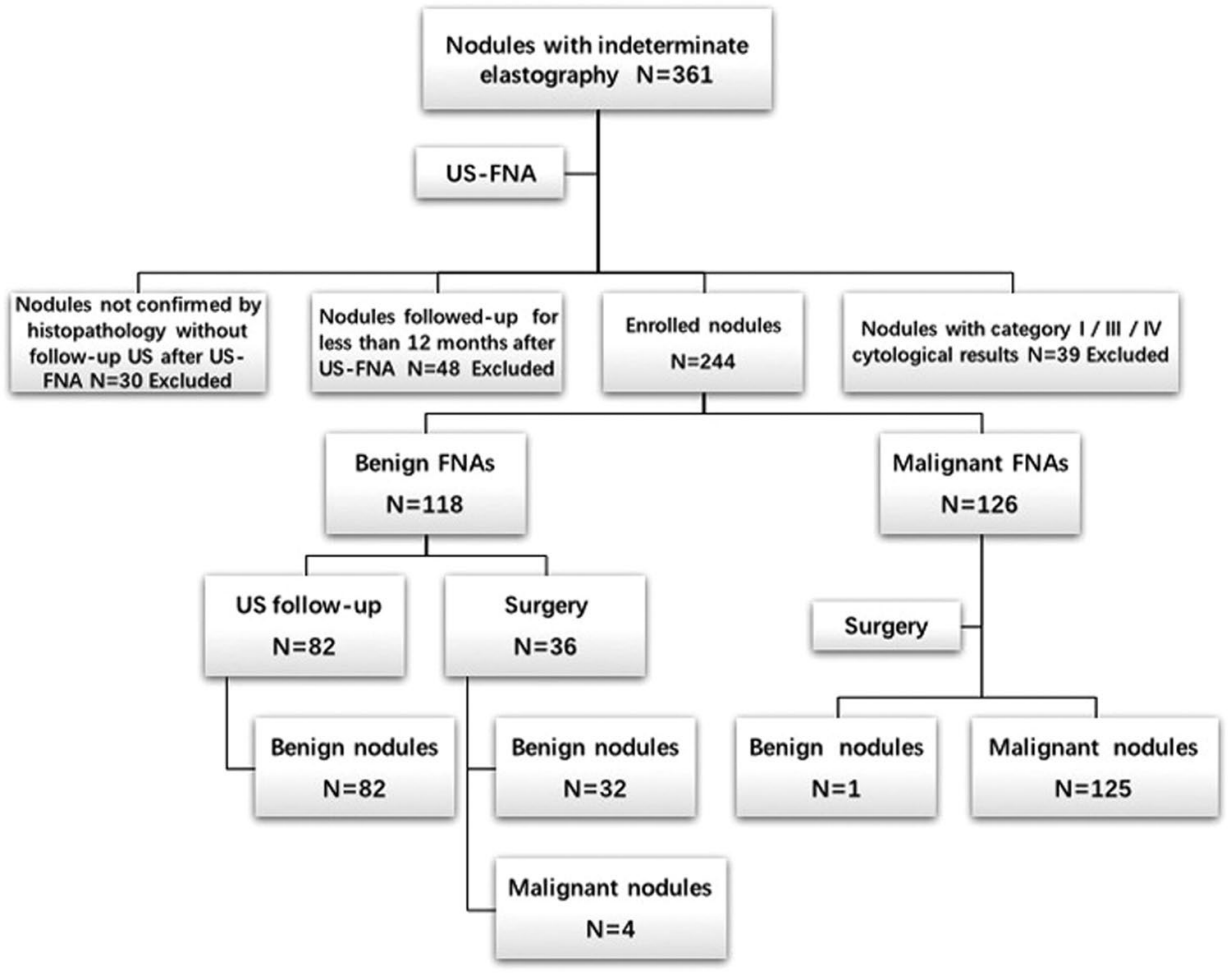
Diagnostic flowchart for all thyroid nodules.

We retrospectively collected the clinical data of 568 thyroid nodules subject to RTE and US-FNA after conventional US examinations between January 2012 to December 2014, and 361 nodules were included in our study. Inclusion criteria were as follows: (1) nodules with indeterminate elastography due to rather large or small size (larger than 3 cm or less than 5 mm), deep location, isthmic or paratracheal position, calcification, and background of thyroiditis (Fig. 1a–e); (2) nodules with indeterminate elastography due to conflicting results between conventional US and RTE (Fig. 1f, for example, a nodule has a clear border and regular margin but significant stiffness). 117 nodules were then excluded from the study according to the following exclusion criteria: (1) nodules not confirmed by histopathology without follow-up US after US-FNA (n = 30); (2) nodules not histologically confirmed that underwent follow-up US examinations for less than 12 months after US-FNA (n = 48); (3) nodules with category I/III/IV cytological results for they could not be definitely classified as benign or malignant by cytological diagnosis (n = 39) (Fig. 2). (The Bethesda system for reporting thyroid cytopathology includes six categories: (I) non-diagnostic or unsatisfactory, (II) benign, (III) atypia of undetermined significance or follicular lesion of undetermined significance, (IV) follicular neoplasm or suspicious for follicular neoplasm, (V) suspicious for malignancy, and (VI) malignant^8^. Among them, categories I, III, and IV are unable to provide a definitive diagnosis).

In total, 244 thyroid nodules (nodule size range, 2.9–49 mm; mean size, 10.3 mm) in 202 patients (150 women and 52 men; age range: 21–81 y; mean age, 45.5 y) were included in this study. The study was approved by Ethics Committee of Shanghai 6th People’s Hospital. All procedures performed in the study involving human participants were in accordance with the ethical standards of the institutional research committee and the 1964 Helsinki declaration and its later amendments or comparable ethical standards. Formal consent is not required for this type of study.

### Conventional US

Thyroid US and Doppler US were performed using the ACUSON S2000 ultrasound system (Siemens, Erlangen, Germany) equipped with a linear probe (4–9 MHz). All patients were examined by one radiologist blinded to the final diagnosis with over 20 years of experience in thyroid sonography.

First, the patient was told to lie down in a supine position with the neck slightly hyperextended, and sonographic features, including border (clear or obscure), margins (regular or irregular), echogenicity (hypoechoic, hyperechoic, isoechoic, and echocomplex), echohomogeneity (homogeneous or inhomogeneous), and microcalcification (absent or present), of the target thyroid nodule were examined. Vascularization was classified according to vascular degree (none, low, medium, and high) and distribution (none, peripheral, and internal). US or clinical signs of thyroiditis were also reported for each patient.

### Real-time elastography (RTE)

In the second part of the examination, elastography was performed by the same radiologist using a US machine (Vision Preirus; Hitachi Medical, Tokyo, Japan) equipped with a 6 to 13 MHz linear transducer. RTE was performed by applying repeated light pressure to the target lesion after the activation of the elastographic mode. The ultrasound probe was used to slightly compress the tissue in the longitudinal axis with a pressure value of 3–4 on the numeric pressure scale (range 1 to 5). Elastography images of thyroid nodules could be classified using a scale of 1–5 according to the colour distribution. Generally, blue indicates “hard-malignancy”, whereas green indicates “soft-benignity”^7^. Scoring was not utilized in this study because all the included nodules obtained indeterminate diagnoses from RTE for several aforementioned confounding factors.

### US-guided FNA

US-guided FNA was performed by another skilled radiologist with over 5 years of experience in thyroid FNA at our institution. Each thyroid nodule was aspirated more than twice using a 25 gauge needle attached to a 10 ml syringe, and an experienced cytopathologist was also present to perform preliminary examination to ensure abundant specimens for a subsequent cytological diagnosis. In our study, cytological results of category II were considered negative, whereas category V/VI was considered positive.

For all nodules, the final diagnosis of malignancy was determined via histopathology of a surgical specimen. Regarding benignity, nodules were considered benign if they were confirmed by histopathology or they exhibited no obvious change on the follow-up sonographic pattern (>12 months) after initial benign cytopathologic results (if surgeries were not performed)^5,14,15^.

### Statistical analysis

The statistical analyses were performed using a statistical package (SPSS 19.0, Chicago, IL, USA). The chi-squared test and Fisher’s exact test were used to compare differences in sonographic features and cytological results between benign and malignant thyroid nodules. The sensitivity (Se), specificity (Sp), positive predictive value (PPV), negative predictive value (NPV) and accuracy of both US-FNA and conventional US in the detection of malignant nodule were calculated. Odds ratios (OR) with 95% confidence intervals (CI) were calculated to determine the malignancy risk of each single parameter. In all cases, p < 0.05 was considered to indicate statistical significance.

## Results

In total, 129 nodules had a final histological diagnosis of malignancy. Among them, 128 nodules were papillary thyroid carcinomas, and 1 nodule was a primary thyroid malignant lymphoma. Of the remaining 115 benign nodules, 36 nodules were histologically confirmed from surgery because another concomitant malignant nodule was present. On the basis of benign cytology, 79 nodules not histologically confirmed underwent US follow-up examinations of over 12 months after US-FNA and exhibited no change in US findings during the follow-up period. Benign nodules included nodular hyperplasia in 41 cases, follicular adenomas in 30 cases, and thyroiditis in 44 cases.

According to the potential influencing factors on indeterminate elastography, all included nodules were divided into six types: large or small size (n = 27, larger than 3 cm/less than 5 mm), deep location (n = 16), isthmic or paratracheal location (n = 30), calcification (n = 65), thyroiditis (n = 44), and conflicting results between conventional US and RTE (n = 62). The rate of cancer prevalence was 74.1%, 75.0%, 73.3%, 46.2%, 27.3%, and 53.2%, respectively (Table 1).

**Table 1:** Influencing factors on indeterminate elastography.

Factors	Number	Prevalence of malignancy (%)
Large or tiny size	27	74.1 (20/27)
Deep location	16	75.0 (12/16)
Isthmic or paratracheal position	30	73.3 (22/30)
Calcification	65	46.2 (30/65)
Thyroiditis	44	27.3 (12/44)
Conflicting results	62	53.2 (33/62)
Total	244	52.9 (129/244)

As shown in Table 2, malignant nodules were more often present with obscure border, irregular margin, inhomogeneous hypoechogenicity and microcalcification. These sonographic features were significantly different between benign and malignant thyroid nodules (p < 0.05). In contrast, no statistical significance was identified regarding vascular degree or distribution (p > 0.05). Se, Sp, PPV, NPV, diagnostic accuracy, and OR were calculated for each significant feature (Table 3). Inhomogeneous echo was the most sensitive feature, but it has the lowest specificity with an OR of 2.31–45.7. Other features, including obscure border, irregular margin, and hypoechogenicity, exhibited similar diagnostic performance with good sensitivity but unsatisfactory specificity and accuracy. However, the opposite result was noted for microcalcification with the best specificity of 73% and the lowest sensitivity of 47.3%. The diagnostic accuracy of sonographic features suspicious for malignancy remained at a middle level from 58.6% to 64.8%.

**Table 2:** Sonographic features of thyroid nodules with indeterminate elastography.

Sonographic features	Benign (n = 115)	Malignant (n = 129)	Total (n = 244)	p Value
Border				0.001
Clear	59	38	97	
Obscure	56	91	147	
Margin				0.000
Regular	55	26	81	
Irregular	60	103	163	
Echogenicity				0.000
Hypoechoic	91	122	213	
Isoechoic				
Hyperechoic	24	7	31	
Echocomplex				
Echohomogeneity				0.000
Homogeneous	16	2	18	
Inhomogeneous	99	127	226	
Microcalcification				0.001
Present	31	61	92	
Absent	84	68	152	
Vascular degree				0.813
None	29	26	55	
Low	57	68	125	
Medium	2	3	5	
High	27	32	59	
Vascular distribution				0.275
None	29	26	55	
Peripheral	8	16	24	
Internal	78	87	165

**Table 3:** Cytological results of thyroid nodules with indeterminate elastography.

Cytological results	Benign	Malignant	Total	P Value
category II	114	4	118	0.000
category V/ VI	1	125	126	
Total	115	129	244	

On cytology, there were 118 nodules with category II (benign), 31 with category V (suspicious for malignancy), and 95 with category VI (malignant) (Table 3). Among them, four papillary carcinomas were misdiagnosed as category II, whereas one adenoma was misdiagnosed as category V. The Se and Sp values of US-FNA were 96.9% and 99.1%, respectively. The PPV and NPV were 99.2% and 96.6%, respectively, with an accuracy up to 98.0%. The data are statistically significant (p < 0.05) with an OR value of 3563 (95% CI 392.4–32344) (Table 4).

**Table 4:** Diagnostic performance of US-FNA and Conventional US.

Characteristic	Se (%)	Sp (%)	PPV (%)	NPV (%)	Accuracy (%)	OR (95% CI)
Obscure border	70.5	51.3	61.9	60.8	61.5	2.52 (1.49–4.27)
Irregular margin	79.8	47.8	63.2	67.9	64.8	3.63 (2.07–6.39)
Hypoechogenicity	94.6	20.9	57.3	77.4	59.8	4.60 (1.90–11.1)
Inhomogeneous echo	98.4	13.9	56.2	88.9	58.6	10.3 (2.31–45.7)
Microcalcification	47.3	73.0	66.3	55.3	59.4	2.43 (1.42–4.16)
FNA	96.9	99.1	99.2	96.6	98.0	3563 (392.4–32344)

Se sensitivity, Sp specificity, PPV positive predictive value, NPV negative predictive value, OR odds ratio, 95% CI 95% confidence interval.

## Discussion

Recent studies have demonstrated that elastography can be a useful adjunct tool for conventional US in the differential diagnosis between benign and malignant thyroid nodules^11,16^. However, specialists from different countries have been aware of the limits of RTE in the application of routine practice^13,17^. Regarding size, nodules larger than 3 cm lack surrounding reference parenchyma, whereas nodules less than 5 mm are too small (Regarding nodules that were too small to have significance, we reported the following information: the study included 19 nodules less than 5 mm, and 12 of them were malignant as confirmed by surgery. Moreover, ten out of the 12 were multifocal carcinomas, in which RTE failed to provide definite diagnoses). Hence, both of these nodules often fail to acquire satisfactory RTE images (Fig. 1a). Nodules with deep locations exhibit less deformation as the corresponding stress transmission is reduced (Fig. 1b). Calcification and thyroiditis within the lesion increase tissue stiffness. All of these conditions could result in artefactual hardening^12,18,19^ (Fig. 1d,e). In addition, for nodules located in improper positions (isthmic or paratracheal), it is often difficult to apply uniform pressure to obtain stable elastographic images^11,20^ (Fig. 1c). Furthermore, conflicting results between conventional US and RTE (e.g., a nodule was inclined to be benign based on sonographic features, such as clear border and regular margin, although its elastographic category indicated malignancy) also confound the final diagnosis (Fig. 1f). Numerous recent studies on RTE have referred to its limitations, but few studies have attempted to identify solutions instead of excluding these cases from their relevant studies^12,20^.

In our study, among nodules sorted by the aforementioned factors, those with calcification and contradiction accounted for approximately half (127/244) with malignancy rates of 46.2% and 53.2%, respectively. Considering the high risk of malignancy, further diagnostic tests to refine indeterminate diagnosis of RTE are necessary to help clinicians select appropriate patient populations requiring surgical intervention.

Conventional US is recognized as the diagnostic fundamental of thyroid nodules. Indeed, our study revealed that most of the suspicious sonographic features (obscure border, irregular margin, marked hypoechogenicity, and microcalcification) had a relatively high sensitivity but low specificity and predictive value. In contrast, microcalcification had a low sensitivity but high specificity. Given that several studies expressed different opinions about the diagnostic value of vascularization, its statistical significance was not observed in our study. This finding is consistent with the conclusion from Frates’s and Rosario’s investigations^21–24^. Therefore, it is still difficult to distinguish between malignant and benign thyroid nodules with indeterminate diagnosis in RTE via a second assessment with conventional US alone.

To date, other diagnostic tests, including FNA, core-needle biopsy, radionuclide scanning, and genetic tests, have been proposed to predict the malignancy of thyroid nodules. Among them, FNA is universally considered as the most simple, safe, accurate, and cost-effective diagnostic tool for unselected nodules. In the current study, 126 nodules with cytological category V/VI exhibited a 3563-fold increased risk of malignancy (OR). US-FNA still provided excellent diagnostic performance (Se 96.9%, Sp 99.1%, PPV 99.2%, NPV 96.6%, and accuracy 98.0%) in the selected nodules of indeterminate elastography despite the presence of several confounding factors (large or small size, calcification, or thyroiditis). Nevertheless, we admit that the perfect diagnostic performance of FNA could be partly attributed to those nodules without repeat aspiration after initially benign cytology, as repeated FNA has been proven to help reduce the false negative rate of FNA in a recent study^25^. However, from a clinical point of view, periodic follow-up of those nodules with benign results on FNA is acceptable due to the indolent nature of thyroid tumours. Many organizations, including the ATA, have also recommend that clinical follow-up is feasible, and further immediate diagnostic studies or treatment are not required if the nodule does not exhibit signs of growth^5,26^.

The indeterminate categories (category III/IV) were not included in this study to assure that all of the included lesions had a definite cytological diagnosis. However, the identification of malignant follicular neoplasm with soft texture via conventional US, RTE, and US-FNA remains challenging, and these lesions can only be defined by the histological findings of vascular or capsular invasion to date^27^. In addition, the high number of malignant thyroid nodules (129/244, 52.9%) was due to the selection bias that only malignancy assessed by surgical pathology could be included in this study.

## Conclusions

Given that the presence of indeterminate RTE is inevitable with a rather high malignant risk due to several confounding factors as demonstrated in our research, thyroid nodules in similar cases should undergo further assessment of malignancy risk. Our study revealed that US-FNA was a valuable tool in nodules with indeterminate elastography and could help clinicians make the most appropriate decision by increasing the detection rate of malignancy.
